# SARS-CoV-2 spike protein S1 subunit induces potent neutralizing responses in mice and is effective against Delta and Omicron variants

**DOI:** 10.3389/fpls.2023.1290042

**Published:** 2023-11-14

**Authors:** Tarlan Mamedov, Damla Yuksel, Irem Gurbuzaslan, Burcu Gulec, Gulshan Mammadova, Aykut Ozdarendeli, Shaikh Terkis Islam Pavel, Hazel Yetiskin, Busra Kaplan, Muhammet Ali Uygut, Gulnara Hasanova

**Affiliations:** ^1^ Department of Agricultural Biotechnology, Akdeniz University, Antalya, Türkiye; ^2^ Institute of Molecular Biology and Biotechnologies, Ministry of Science and Education of Azerbaijan, Baku, Azerbaijan; ^3^ Department of Microbiology, Medical Faculty, Erciyes University, Kayseri, Türkiye; ^4^ Vaccine Research, Development and Application Center, Erciyes University, Kayseri, Türkiye

**Keywords:** plant transient expression system, SARS-CoV-2, S1 subunits of spike protein, delta, Omicron variants

## Abstract

SARS-CoV-2, the virus responsible for the COVID-19 pandemic, belongs to the betacoronavirus genus. This virus has a high mutation rate, which rapidly evolves into new variants with different properties, such as increased transmissibility or immune evasion. Currently, the most prevalent global SARS-CoV-2 variant is Omicron, which is more transmissible than previous variants. Current available vaccines may be less effective against some currently existing SARS-CoV-2 variants, including the Omicron variant. The S1 subunit of the spike protein of SARS-CoV-2 has been a major target for COVID-19 vaccine development. It plays a crucial role in the virus’s entry into host cells and is the primary target for neutralizing antibodies. In this study, the S1 subunit of the spike protein of SARS-CoV-2 was engineered and produced at a high level in *Nicotiana benthamiana* plant. The expression level of the recombinant S1 protein was greater than the 0.5-g/kg fresh weight, and the purification yield was at least ~0.3 g of pure protein/kg of plant biomass, which would make a plant-produced S1 antigen an ideal vaccine candidate for commercialization. Purified, the plant-produced SARS-CoV-2 S1 protein exhibited significantly higher binding to the SARS-CoV-2 receptor, angiotensin-converting enzyme 2 (ACE2). Moreover, we also show that recombinant S1 protein/antigen-elicited antibodies can neutralize the Delta or Omicron variants. Collectively, our results demonstrate that a plant-produced S1 antigen could be a promising vaccine candidate against SARS-CoV-2 variants including Omicron.

## 1 Introduction

COVID-19, caused by the SARS-CoV-2 virus, has had a significant impact on people and economies worldwide. Since its emergence in late 2019, the virus has rapidly spread across the globe, leading to a pandemic that has affected nearly every aspect of life. The virus has spread rapidly and led to millions of infections and nearly 7 million deaths worldwide. According to the Worldometer report (https://www.worldometers.info/coronavirus/), there are still around 250 deaths per day from SARS-CoV-2 infection. SARS-CoV-2 had a natural tendency to mutate and give rise to new variants over time. Several variants of SARS-CoV-2 such as Alpha (B.1.1.7), Beta (B.1.351), Gamma (P.1), Delta (B.1.617.2), and Omicron (B.1.1.529) have been identified since the beginning of the COVID-19 pandemic. These variants have exhibited differences in terms of transmissibility, disease severity, and immune evasion. The Omicron variant of SARS-CoV-2 is the dominant strain of SARS-CoV-2 and currently accounts for more than 98% of cases worldwide. Several vaccines, which have been authorized, approved, and used against SARS-CoV-2, have shown efficacy in preventing COVID-19 and in reducing the severity of the disease. However, existing vaccines have been shown to be less effective or ineffective against specific mutations or variants of SARS-CoV-2. Different variants, such as the Delta and Omicron variants, have raised concerns due to their potential to increase transmissibility and potentially evade certain immune responses.

Since SARS-CoV-2 has the ability to mutate over time, which can lead to the emergence of new variants, it is possible that these variants might be more severe or vaccine-resistant. Therefore, developing effective COVID-19 vaccines and adopting strategies to control the spread of the virus are very important. In other words, the development of vaccines effective against emerging variants, such as Delta, Omicron, or possible future variants, is still of high priority and urgently needed. In this study, we produced S1 subunits of the SARS-CoV-2 spike protein in the *Nicotiana benthamiana* plant, with the results described for the first time in our prepublished report in bioRxiv (Mamedov et al., 2020). It should be noted that the majority of the epitopes targeted by neutralizing antibodies are found on the spike protein of the SARS-CoV-2 virus. The spike protein is responsible for facilitating the entry of the virus into host cells by binding to the ACE2 receptor on the cell surface (Lan et al., 2020; Shang et al., 2020; Walls et al., 2020; Zhou et al., 2020; Jackson et al., 2022). The spike protein of SARS-CoV-2 consists of S1 and S2 subunits. The S1 subunit is primarily involved in receptor binding and is a major target for neutralizing antibodies (Dong et al., 2020; Huang et al., 2020). Most COVID-19 vaccines currently available focus on eliciting an immune response against the S1 protein.

Plant-based production systems offer several advantages over traditional microbial or mammalian expression systems, such as scalability, low production costs, ease of cultivation, reduced risk of contamination with animal pathogens, and eukaryotic-type posttranslational modifications that are similar to those in human cells. Plant expression systems have been successfully utilized for the cost-effective and rapid production of wide-range recombinant proteins, such as vaccine candidates (Farrance et al., 2011; Mamedov et al., 2012; Tottey et al., 2018; Wang et al., 2019; Shanmugaraj et al., 2020; Tottey et al., 2023; Mamedov and Yusibov, 2013; Mamedov et al., 2017; Mamedov et al., 2019a; Mamedov et al., 2019b; Mamedov et al., 2021a; Mamedov et al., 2021b; Mamedov et al., 2021c; Mammadova et al., 2022), therapeutic proteins, enzymes (Mamedov et al., 2021c), monoclonal antibodies, clotting (Mamedov et al., 2019a), and growth factors and other valuable products. The plant expression system has been used for production several vaccine candidates against COVID-19 (Capell et al., 2020; Shanmugaraj et al., 2020; Gobeil et al., 2021; Mamedov et al., 2021a; Mamedov et al., 2021b; Royal et al, 2021; Shanmugaraj et al., 2021; Siriwattananon et al., 2021; Ward et al., 2021; Hager et al., 2022; Moon et al., 2022; Pillet et al., 2022; Ruocco and Strasser, 2022; Mamedov et al., 2023; O'Kennedy et al., 2023). However, with the exception of our recent work (Mamedov et al., 2023), there has been no report of efficacy of these candidate vaccines against the Omicron variant. We demonstrated that plant-produced RBD and cocktail-based vaccine candidates are highly effective against SARS-CoV-2, independently of its emerging variants (Mamedov et al., 2023). This was the first and only report worldwide on a COVID-19 vaccine that is effective for all emerging variants of SARS-CoV-2, including Delta and Omicron.

In this work, we show that another COVID-19 vaccine candidate, the full-length S1 subunit of SARS-CoV-2, that is produced at high levels in *N. benthamiana* plant, elicits protective immune responses in mice. In addition, mice immunized with the S1 subunit of SARS-CoV-2 demonstrated significant neutralizing activity against Delta and Omicron variants.

## 2 Materials and methods

### 2.1 Engineering, cloning, and production of S1 protein in *N. benthamiana*

After codon optimization (using *N. benthamiana* plant codons), the gene of the S1 domain of the spike protein of SARS-CoV-2 (AA 14-815, GenBank accession MN985325), was *de novo* synthesized (Biomatik Corp., Canada). The protein was expressed as a His6 tag to facilitate the purification of plant-produced S1 protein. The signal peptide of the PR1a protein of *Nicotiana tabacum* (MGFVLFSQLPSFLLVSTLLLFLVISHSCRA) was placed at the N-terminal of S1 protein. The endoplasmic reticulum (ER) retention signal (KDEL) was placed C-terminus after the S1protein sequence. The S1-His6-KDEL sequence was cloned into the pEAQ (Sainsbury et al., 2009) plant expression vector to generate pEAQ-S1-His6-KDEL. This pEAQ-S1-His6-KDELconstruct was then transferred into Agrobacterium strains, AGL1 and EHA105, by electroporation. Transformed *A. tumefaciens* strains AGL1 and EHA105 harboring pEAQ-S1-His6-KDEL constructs were grown overnight at 28°C in BBL medium containing 5 g/l yeast extract, 5 g/L NaCl, 10 g/l soy hydrolysate, and 50 mg/l kanamycin. Agrobacterium cultures grown overnight were then infiltrated into 6- to 7-week-old *N. benthamiana* plant leaves by manual infiltration using a syringe. Infiltrated *N. benthamiana* leaves were harvested 4–8 days after post infiltration (dpi) for protein expression analysis by Western blot or ELISA.

### 2.2 Purification of S1 protein from *N. benthamiana* leaves

The S1 protein was purified from *N. benthamiana* leaves by nickel column affinity chromatography using HisPur™ Ni-NTA Resin (Thermo Fisher Scientific, Cat. No. 8822). Frozen plant leaves were ground in a 20 mM sodium phosphate extraction buffer (containing 10 mM imidazole and 300 mM sodium chloride, pH 7.4) in a volume of three times the leaf weight. Homogenized cell extract was filtered through Miracloth and then centrifuged at 20,000 g for 25 min. The resulting extract was filtered using a 0.45-µm syringe filter (Millipore). The filtered supernatant was loaded onto HisPur™ Ni-NTA resin equilibrated with an equilibration buffer containing 20 mM sodium phosphate containing 10 mM imidazole and 300 mM sodium chloride, pH 7.4. The Ni-NTA resin column was first washed with 20 mM sodium phosphate, 25 mM imidazole, and 300 mM sodium chloride, pH 7.4 (wash buffer), to elute the unbound protein fraction. The proteins were eluted from the Ni-NTA resin column with 10 CV 20 mM sodium phosphate, pH 7.4, containing 250 mM imidazole and 300 mM sodium chloride (elution buffer). Fractions eluted from the column were collected, combined, and concentrated using a concentrator (Millipore, 10K MWCO). The protein concentration of eluted fractions and concentrated fractions were determined by BioDrop. The concentrated fraction was finally exchanged against the PBS buffer and stored at –80°C until use.

### 2.3 Gel filtration

Gel filtration chromatography analysis was performed using the AKTA start system to obtain the S1 protein purified by affinity chromatography with greater purity. A HiPrep 10/40 column and Sephacryl S-200 High Resolution (HR) resin (GE Healthcare, WI, USA) were used in the device. The program was adjusted to a column volume of 35 ml, a flow rate of 2.0 ml/min, and a pressure limit of 0.3 MPa. Approximately 250 µg of S1 protein was loaded into the equilibrated system with 50 mM sodium phosphate buffer, pH 7.4, and 50 mM sodium chloride. Fractions were pooled using the same buffer for elution, and an elution profile was obtained. The concentrations of the elution fractions were measured with BioDrop, and the fractions in the pick part in the profile were combined and concentrated with a Millipore concentrator (10K MWCO). The concentrated protein was analyzed by SDS-PAGE and Western blot analysis.

### 2.4 Stability analysis

The S1 protein produced in *N. benthamiana* plant was subjected to time and temperature stability analysis. For time- and temperature-dependent stability analyses, S1 protein, purified from the *N. benthamiana* plant, was diluted to 1.0 mg/ml in low-binding polypropylene Eppendorf tubes. The S1 protein was subjected to stability analysis at 37°C for 96 h at 24-h intervals. After incubation, samples were mixed with SDS loading dye and stored at −80°C until analyzed by SDS-PAGE and Western blot.

### 2.5 ELISA

ELISA was performed as described recently (Mamedov et al., 2021a; Mamedov et al., 2021b; Mamedov et al., 2023). The plates (Greiner Bio-One GmbH, Germany) were coated with 100 ng of recombinant S1 protein, purified from *N. benthamiana* leaves using Ni-column affinity chromatography, and incubated at 4°C overnight. After incubation, the plates were blocked with blocking buffer at room temperature for 2 h. After blocking, commercial and plant-produced gACE2 and dACE2 were added into wells in different concentrations (5.5 nM, 11.0 nM, 55.0 nM, 110.0 nM, and 220.0 nM concentrations for commercial ACE2, 1.33 nM, 2.25 nM, 5.0 nM, 27.5 nM, 55.0 nM, and 110.0 nM concentrations for plant-produced gACE2 and dACE2) and incubated at 37°C for 3 h. After 3 h, human anti-ACE or anti-His tag mAb (Cat. no. 6525 05, BioLegend) or purified anti-human ACE2 monoclonal antibodies (Cat. no. 375801, BioLegend, CA) were added to each well. After washing the plates three times with 200 µl/well blocking solution, the wells were treated with anti-rat (Cat. no. MBS440123), anti-mouse (Cat. no. 405306, BioLegend), or anti-human IgG antibody (Cat. no. MBS440121, MyBioSource). After the plates were washed three times with 1× PBS-T wash solution (200 µl/well for 5 min), an OPD substrate tablet (o-phenylenediamine dihydrochloride) (Sigma) was added to wells (200 µl/each). The plate was then incubated for 30 min at room temperature (in the dark), and after the incubation, the plates were read at 450 nm in a multiwell plate reader.

### 2.6 Immunogenicity studies in mice for S1 protein

For immunogenicity studies, 7-week-old *BALB/c* mice (male, six animals per group) were intramuscularly immunized with plant-produced S1 antigen adsorbed to 0.3% Alhydrogel at 0 and 21 days. Blood samples were collected from immunized mice on days 21 and 42. In the control group, mice were immunized with 1× PBS with Alhydrogel. Serum samples, collected on days 21 and 42, were evaluated for anti-S1 domain antibody responses with an IgG ELISA. IgG titers of S1 protein-injected sera were determined by ELISA as described previously (Mamedov et al., 2021a; Mamedov et al., 2021b). Briefly, a 96-well plate (Greiner Bio-One GmbH, Germany) was coated with 200 ng of plant-produced S1 protein, diluted in 100 mM carbonate coating buffer, and incubated for overnight at 4°C. After blocking the plates with blocking buffer, different dilutions of mouse serum (10^2^ to 10^8^) collected from S1 protein-injected mice were added and incubated for 2 h at 37°C. After 2 h, the plate was first washed with blocking solution (200 µl/well) for three times and then the wells were treated with anti-mouse IgG antibody. After washing three times with 1× PBS, the plates were then washed with 1× PBST (200 µl/well for 5 min) and then 200 µl of substrate solution (Sigma) was added to wells. After incubating the plates at room temperature for 30 min in the dark, the plates were read at 450 nm in a plate reader. Immunogenicity studies were carried out under the supervision of a veterinarian at the Akdeniz University Experimental Animal Care.

### 2.7 Glycan detection

The confirmation of the presence of glycans in the purified plant-produced gS1 protein and *in vivo* enzymatic removal of N-glycans in plant-produced dS1 protein was performed as described recently (Mamedov et al., 2021a; Mamedov et al., 2021b). Briefly, 0.25 µg of gS1 protein, *in vivo* deglycosylated S1 (dS1) protein, and *in vitro* deglycosylated S1 proteins was run on a 10% SDS-PAGE and then presence or removal of glycans in the plant-produced S1 proteins on the gel was confirmed using the Pro-Q Emerald Glycoprotein Stain Kit (Catalog number, P21857 Thermo Fisher Scientific). The stained proteins in the gel were visualized on a UV illuminator.

### 2.8 Microneutralization assay

An *in vitro* microneutralization assay was performed as described recently (Mamedov et al., 2021a; Mamedov et al., 2021b; Mamedov et al., 2023). Briefly, mouse sera immunized with plant-produced S1 protein and collected on day 42 were tested against live SARS-CoV-2 variants, Wuhan, Delta, and Omicron, in the Vero E6 cell line. A neutralizing antibody specific for SARS-CoV-2 was determined using a microneutralization test (MNT). Before use, serum samples were heat-inactivated for 1 h at 56°C. Sera samples, which were diluted in 1/8-1/1024, were incubated with the SARS-CoV-2 variants of Wuhan, Delta, and Omicron (100 TCID50) at 37°C for 1 h, and the procedures described recently were followed (Mamedov et al., 2021b; Mamedov et al., 2023).

### 2.9 Statistical analysis

The statistical analysis was performed as described recently (Mamedov et al., 2021a; Mamedov et al., 2023). For all statistical analyses, GraphPad Prism software was used. One-way ANOVA test was used (i) to examine the binding affinity of the gS1 protein of SARS-CoV-2 to ACE2, (ii) to compare the antibody responses of sera inoculated with gS1 protein, and (iii) to test the neutralization ability of sera from mice vaccinated with plant-produced gS1 protein against infection of the live SARS-CoV-2 variants.

## 3 Results

### 3.1 Engineering, cloning, expression, production, and purification of S1 of SARS-CoV-2 from the *N. benthamiana* plant

**Figure 1:**
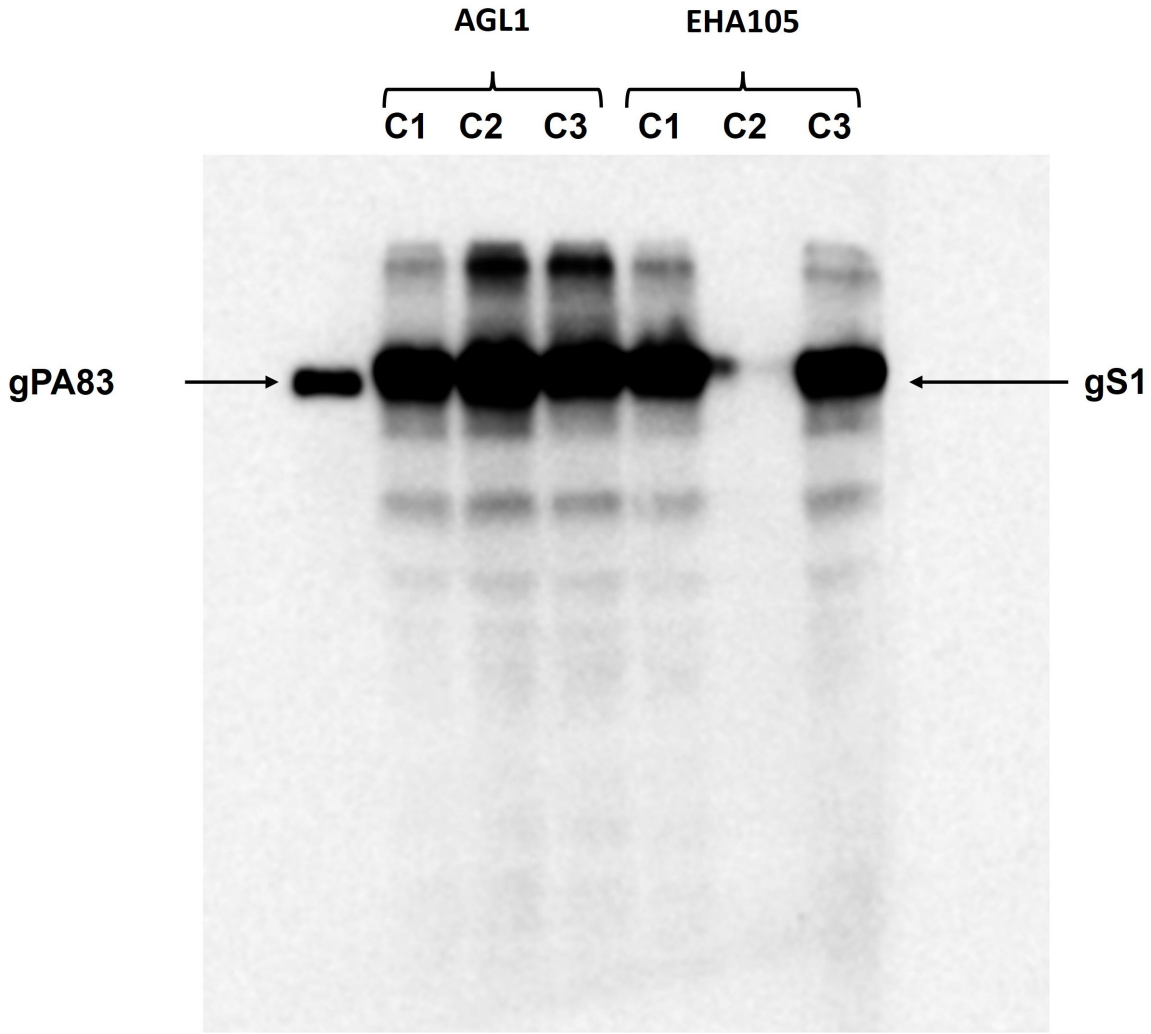
Western blot analysis of the expression of the gS1 protein produced in the plant by different *Agrobacterium* strains. The pEAQ-S1 gene was introduced into both *A. tumefaciens* AGL-1 and EHA105 strains by agroinfiltration. Leaves infiltrated with gS1 protein were harvested at 5 dpi. The plant-produced gPA83 protein was loaded as a control protein (25 ng).

**Figure 2:**
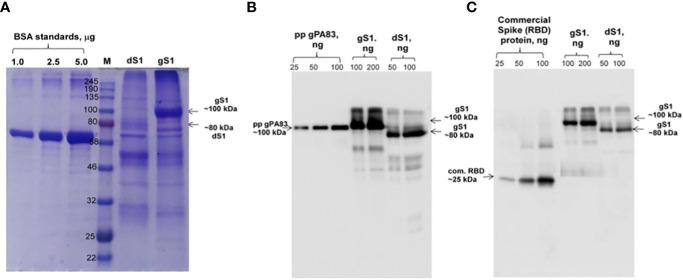
SDS-PAGE **(A)** and Western blot **(B, C)** analysis of purified plant-produced S1 protein. The S1 variant from the *N. benthamiana* plant was purified using HisPur™ Ni-NTA resin. Membranes were probed with anti-His tag monoclonal antibody **(B)** or commercially available anti-RBD antibody (MBS2563840, MyBioSource, USA). M: color-prestained protein standard (NEB). gS1: plant-produced glycosylated S1 protein; dS1: plant-produced deglycosylated S1 protein. Commercial spike protein: COVID 19 Spike Protein (RBD) Active Protein; sequence positions Arg319-Phe541 (Cat. no. MBS2563882, MyBioSource, USA).

Engineering, cloning, expression, production, and purification of S1 protein were performed as described in Materials and Methods. The expression of S1 protein in the *N*. *benthamiana* plant (harvested at 5 dpi) was confirmed by the Western blot analysis using an anti-His tag antibody (**Figure 1**). The *N. benthamiana* leaf samples infiltrated with the pEQA-S1 gene were harvested at different post-infiltration days (dpi) and expression levels of gS1 protein were found to be maximum at 5 dpi. Colony C2 (AGL1) (**Figure 1**, as indicated) was used for further purification of S1 protein. The expression level of gS1 determined by ELISA and Western blot was ~0.5 g/1 kg of plant leaf. Deglycosylated S1 protein was produced by co-expression of the S1 subunit gene with Endo H, as was achieved and described previously (Mamedov et al., 2012; Mamedov et al., 2017; Mamedov et al., 2019b; Mamedov et al., 2021a1b; Mamedov et al., 2021c; Mamedov et al., 2023). Both deglycosylated and glycosylated variants of plant-produced S1 proteins were purified from *N. benthamiana* plant leaves using HisPur™ Ni-NTA resin. On SDS-PAGE and Western blotting, plant-produced glycosylated S1 (gS1) protein appears as a major protein band with an MM of ~100 kDa (**Figures 1**, **2A**). However, with no clear visible band of Ni-NTA resin purified, Endo H-deglycosylated S1 protein (dS1) was observed in SDS-PAGE gels, suggesting large degradation of the deglycosylated S1 protein. On Western blotting, deglycosylated S1 protein with a band of ~80 kDa was observed. Plant-produced, purified glycosylated, and deglycosylated S1 protein variants were recognized by anti-His mAb tag (**Figure 2B**) or anti-S protein specific pAb (**Figure 2C**).

### 3.2 Glycan detection analysis of glycosylated and deglycosylated S1 protein variants

**Figure 3:**
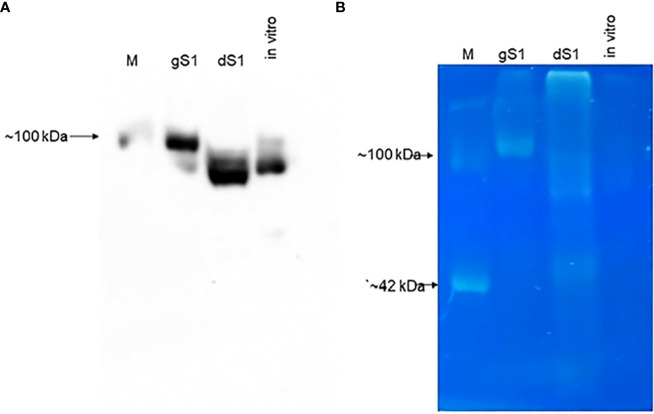
Glycan detection analyzes gS1 and dS1 proteins. **(A)** Western blot analysis of plant-produced gS1 and dS1 and *in vitro* deglycosylation of gS1 protein with commercial Endo H (*in vitro*). M: MagicMark™ XP Western Protein Standard, Catalog no: LC5602, Thermo Fisher Scientific). Images were obtained using the GeneGnome XRQ Chemiluminescence imaging system (Syngene, A Division of Synoptics Ltd.). **(B)** Glycan detection analysis of gS1 and dS1 proteins. Glycan analyses were performed with plant-produced gS1 and dS1 proteins and the *in vitro* deglycosylated version of gS1 protein as indicated (*in vitro*). Glycan detection was performed using the Pro-Q Emerald 300 Glycoprotein Staining Kit (Cat. No. P33378, Molecular Probes, Grand Island). CM, CandyCane glycoprotein molecular weight standards (Molecular Probes).

To demonstrate the *in vivo* deglycosylation of plant-produced S1 protein by Endo H, we conducted the glycan detection analysis of glycosylated and deglycosylated variants of S1 protein as described in Materials and Methods. As shown in **Figure 3B** (as indicated), the glycan was only detected in plant-produced glycosylated S1 protein. **Figure 3A** demonstrates the Western blot analysis of the same samples, diluted 1/10-fold, probed with an anti-His tag antibody.

**Figure 4:**
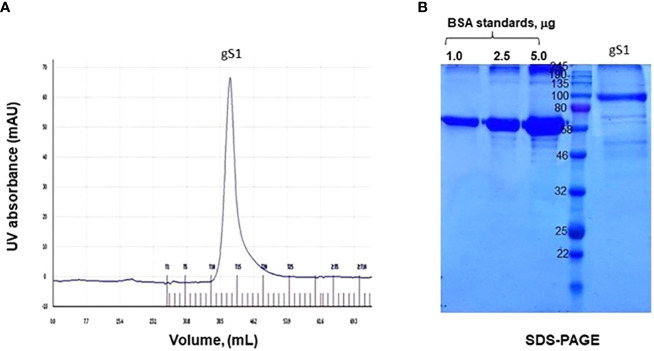
Gel filtration profile of S1 protein and SDS-PAGE analysis of column eluted fraction. gS1 protein was purified by size exclusion chromatography. **(A)** Elution profile of gS1 in the AKTA start system. **(B)** SDS-PAGE analysis for gel-filtered gS1 protein. BSA standards were loaded as 1.0, 2.5, and 5.0 μg.

Plant-produced gS1 protein, purified using HisPur*™* Ni-NTA resin, was subjected to gel-filtration chromatography. The purified S1 protein was loaded onto GE Healthcare Sephacryl TM S-200 High Resolution resin. The gel chromatography step was performed on the ÄKTA Start purification system, and approximately 250 µg of plant-produced, purified S1 protein was loaded into the column. As can be seen from **Figure 4A**, gS1 protein was eluted as a single pick from the Sephacryl S-200 column, and no detectable aggregation was observed. The eluted fraction was combined and analyzed on SDS-PAGE (**Figure 4**). The purity of the S1 protein eluted from the Sephacryl S-200 column was higher than 85%.

### 3.3 Binding affinity of plant-produced S1 protein with ACE2

**Figure 5:**
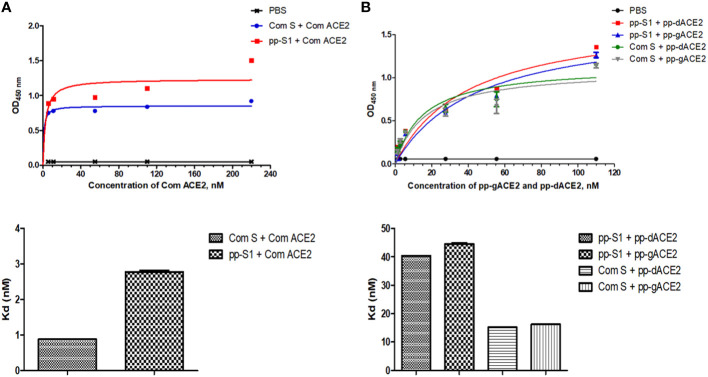
Assessment of binding ability of S1 protein to different ACE2 variants. **(A)** Graph for binding affinity of S1 protein to commercial ACE2. The plate was coated with S1 protein and incubated with commercial ACE2 (cat. no. 792004, BioLegend, USA) and evaluated with an anti-His primary antibody. **(B)** Graph for binding affinity of S1 protein to plant-produced glycosylated ACE2 (gACE2) and deglycosylated ACE2 (dACE2). Plate coated with S1 protein and incubated with plant-produced gACE2 and dACE2. The binding between S1 protein and ACE2 was evaluated using an anti-ACE2 primary antibody (Cat. no. 375801, BioLegend, CA, United States). Commercial spike protein (Cat. no. MBS2563882, MyBioSource, CA, United States) was used as control. Each point on the graph is derived from triplicate for each dilution.

Binding of the plant-produced gS1 protein to ACE2 was performed as described in the Materials and Methods. Commercial ACE2, plant-produced gACE2 and dACE2 proteins were used for the affinity assessment of S1 protein. Commercial spike protein was used as control protein. As seen in **Figure 5**, the S1 protein showed specific binding to the SARS-CoV-2 receptor, ACE2.

### 3.4 Stability assessment of plant-produced gS1 protein

**Figure 6:**
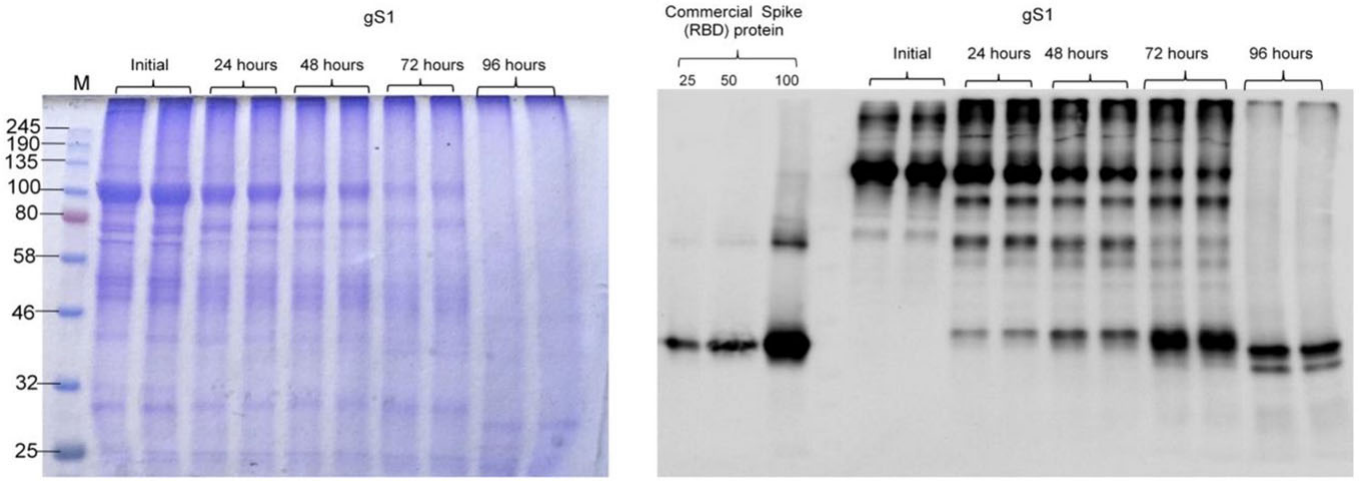
Stability of the glycosylated S1 protein. The plant-produced, Ni-NTA resin affinity column-purified glycosylated S1 protein was incubated at 37°C for 24, 48, 72, and 96 h and analyzed by SDS-PAGE **(A)** and Western blotting **(B)** analyses. Lanes were loaded with ~5.0 μg **(A)** or 200 ng **(B)** S1 proteins. M: color-prestained protein standard. Proteins on the blot were probed with COVID-19 Spike RBD Polyclonal Antibody **(B)**. The image was taken using the highly sensitive GeneGnome XRQ Chemiluminescence imaging system.

The stability assessment of plant-produced glycosylated gS1 of SARS-CoV-2 was examined after incubation at 37°C for 24, 48, 72, and 96 h, and the results are presented in **Figure 6**. The plant-produced gS1 protein degraded by around 50% after 48 h. Since the yield of Endo H dS1 protein was very low, we were not able to compare the stability of the gS1 protein with its dS1 counterpart.

### 3.5 Immunogenicity studies of S1 protein variants in mice

**Figure 7:**
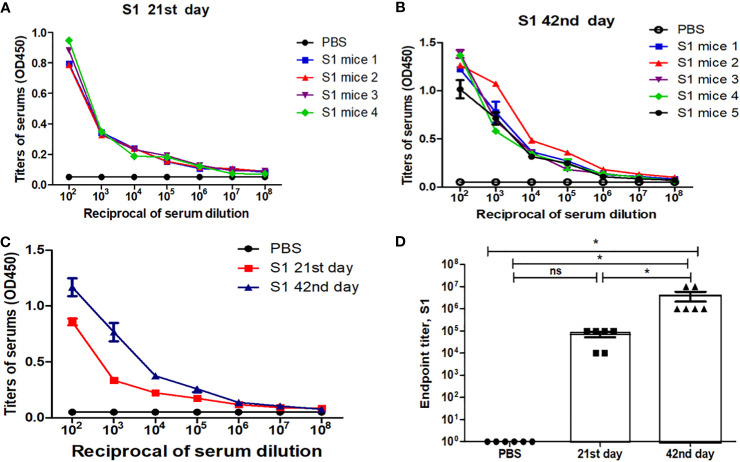
Immunogenicity studies of recombinant S1 protein, purified from *N. benthamiana* plants using Ni-affinity chromatography. Mice were immunized with the S1 protein (5 µg, with Alhydrogel adjuvant), purified from *N. benthamiana* plant, on study days 0 and 21 intramuscular injection (IM). The determination of IgG titers was done by ELISA in sera collected from mice immunized with S1 protein at day 21 **(A)** and day 42 **(B)** postvaccination, and the summary of 21st- and 42nd-day antibody titer is shown in **(C)** Detection of S1-specific endpoint IgG titers presented in **(D)** Endpoint titer refers to the reciprocal dilutions of a serum yielding an average OD value four times greater than pre-immune control samples. The control group received PBS with Alhydrogel. Data are shown as mean ± standard error of the mean of triplicates (SEM) in each sample dilution. *p < 0.05; ns, non significant.

Mice received two doses of 5 µg of the gS1 protein adsorbed on 0.3% Alhydrogel at 3-week intervals (21 days). On days 21 and 42, serum samples were collected and evaluated for anti-gS1 antibody responses. The IgG responses obtained showed that the plant-produced S1 protein was able to induce significantly higher antibody titers with alum adjuvant at a dose of 5 µg (**Figure 7**).

### 3.6 *In vitro* neutralization

**Figure 8:**
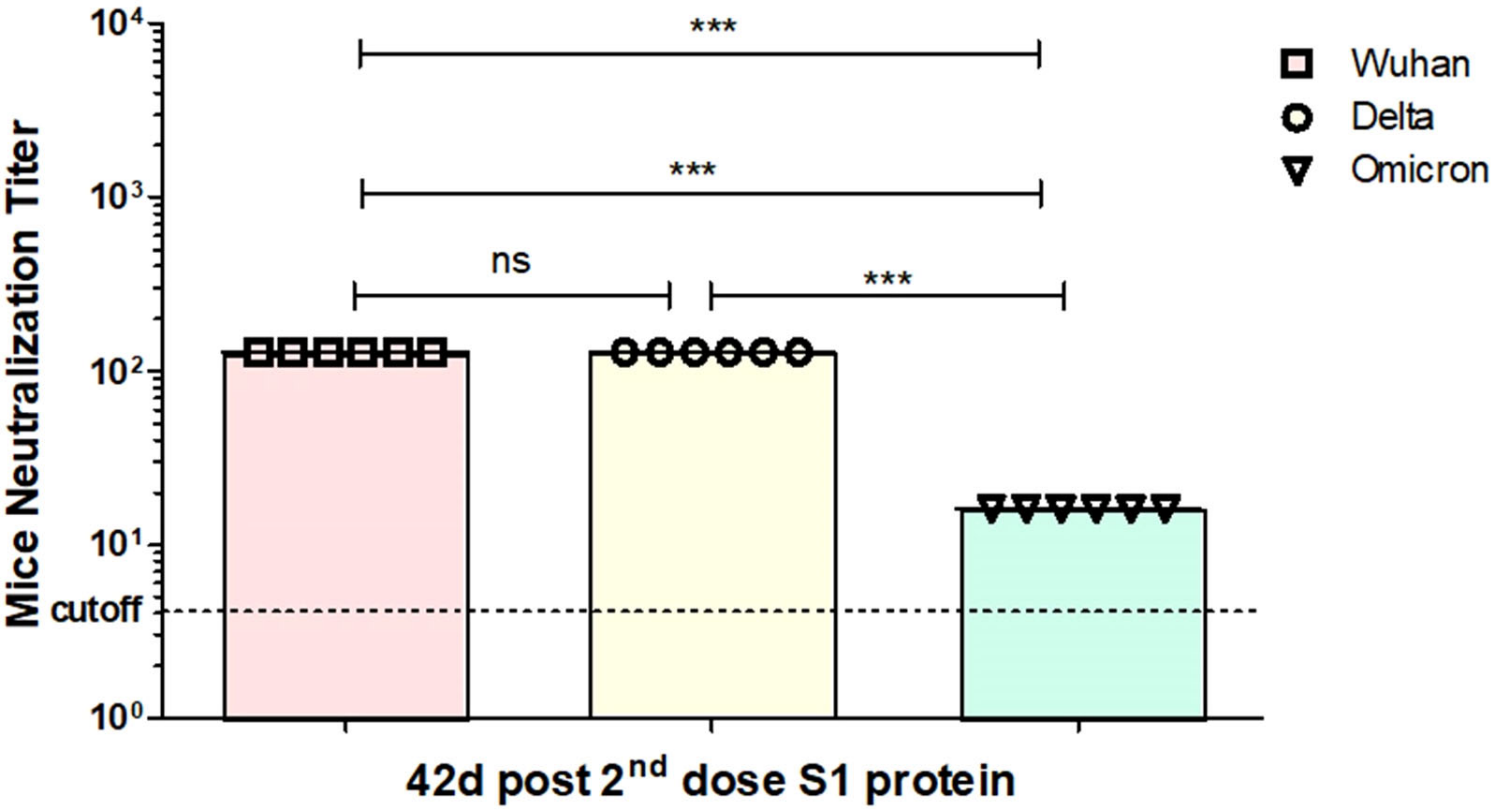
*In vitro* microneutralization assay of plant-produced gS1 protein immunized mouse serum against SARS-CoV-2 variants (live) in the Vero E6 cell line. In the experiment, neutralization ability of gS1 protein was tested against Wuhan, Delta, and Omicron variants of SARS-CoV-2. 32- to 1,024-fold dilutions of mouse sera immunized with gS1 protein were used. Using the t-test statistical significance (p < 0.05) was calculated ***p < 0.001. ns, non significant.

As described earlier, the titers of the Wuhan (GB-MT327745; GISAID-EPI_ISL_424366), Delta (GB-OM945721; GISAID-EPI_ISL_10844545), and Omicron (GB-OM945722; GISAID-EPI_ISL_10844681) variants of SARS-CoV-2 were determined using the tissue culture infective dose 50% (TCID50) method, which measures the amount of virus needed to infect 50% of the cultured cells (Pavel et al., 2020). All experiments with infectious SARS-CoV-2 strains were performed in a biosafety level 3 (BSL3)-enhanced facility at Erciyes University Vaccine Research, Development and Application Center (ERAGEM). *In vitro* neutralization experiment was performed as described recently (Mamedov et al., 2021a; Mamedov et al., 2021b; Mamedov et al., 2023). The neutralization activity of sera from mice immunized with the recombinant S1 protein against live Wuhan (GB-MT327745; GISAID-EPI_ISL_424366), Delta (GB-OM945721; GISAID-EPI_ISL_10844545), and Omicron (GB-OM945722; GISAID-EPI_ISL_10844681) variants of SARS-CoV-2 infection was tested in Vero-E6 cells. Neutralization titers for the S1 protein are shown in **Figure 8**. As can be seen from **Figure 8**, after two doses of the plant-produced S1 protein, neutralizing titers of Omicron variant were reduced around eightfold compared with neutralizing titers of Wuhan (**Figure 8**). At the same condition, neutralizing titers of the Delta variant were similar compared with neutralizing titers of Wuhan.

## 4 Discussion

The S1 subunit of spike protein of SARS-CoV-2 has many neutralizing epitopes; therefore, it is a major target for COVID-19 vaccine development (Ahmed et al., 2020). It plays a crucial role in the virus entry into host cells and is the primary target for neutralizing antibodies (Dong et al., 2020; Huang et al., 2020). All individuals who recovered from COVID-19 infection were shown to develop anti-S1 and anti-RBD antibodies (Song et al., 2022). Since the initial identification of SARS-CoV-2, several variants have already been detected and studied, including the Alpha (B.1.1.7), Beta (B.1.351), Gamma (P.1), Delta (B.1.617.2), and Omicron variants. Neutralizing titers of the Omicron variant were reduced around eightfold compared with neutralizing titers. These variants have exhibited differences in terms of transmissibility, disease severity, and immune evasion. Currently available and used vaccines are less effective or not effective against Omicron variants. SARS-CoV-2 has the ability to mutate over time, and it can lead to the emergence of new variants, and it is possible that it might be more severe or vaccine-resistant. Therefore, developing effective COVID-19 vaccines and adopting new strategies to control the spread of the virus is critical. In other words, the development of vaccines effective against the currently existing variants, such as Delta or Omicron or possible future emerging variants, is still a high priority and urgently needed. Recently, we reported that plant-derived RBD or antigen cocktail (N+RBD) variants are highly effective against emerging SARS-CoV-2 variants, including Delta and Omicron (Mamedov et al., 2023). In this study, we demonstrate that another COVID-19 vaccine candidate, plant-produced S1 protein, could be a promising candidate as an effective vaccine to all emerging variants of SARS-CoV-2. Plant expression systems have shown to be effective platforms for the cost-effective and rapid production of a variety of complex recombinant proteins, such as therapeutic proteins, vaccine candidates, antibodies, enzymes, and other biologics. The ongoing advancements in plant biotechnology continue to expand the possibilities for utilizing plant-based production systems in various applications within the field of biotechnology and medicine. Using plant expression system, RBD and a VLP-based vaccine candidate against SARS-CoV-2 variants were developed by different research groups (Mamedov et al., 2021a; Mamedov et al., 2021b; Royal et al, 2021; Shanmugaraj et al., 2021; Moon et al., 2022; Song et al., 2022; Mamedov et al., 2023; O'Kennedy et al., 2023). A number of plant-derived SARS-CoV-2 RBD-based antigens and VLPs are in preclinical and clinical trials (Gobeil et al., 2021; Shanmugaraj et al., 2021; Ward et al., 2021; Hager et al., 2022; Pillet et al., 2022; Ruocco and Strasser, 2022). A S1 subunit of SARS-CoV-2 fused with the Fc domain was expressed in the *N. benthamiana* plant using the pBYR2e geminiviral plant expression vector. It was shown that mice immunized with purified S1-Fc protein induced antigen-specific antibodies and T-cell responses in mice (Panapitakkul et al., 2022). The expression level, determined by ELISA to be reported as 30 mg/kg of fresh leaf, seems to be low for commercialization. As we described earlier, fusion of the Fc domain to some target proteins may facilitate their production. However, since the fusion adds additional amino acids to the target protein sequence, it significantly affects on protein folding, functional activity, stability, and finally product yield (Andersen et al., 2014; Mamedov et al., 2021c). Production of recombinant proteins with their native-like form is very important as it is critically essential for proper folding, stability, and functional activities of expressed recombinant proteins. The stability of recombinant proteins is important for the high production of target proteins in a plant system, which is one of the key factors for commercialization. The expression level of ACE2-Fc fusion was reported to be around 100 mg/kg in plant leaves. However, the expression levels of the truncated, soluble ACE2s were 7.5 times higher than the ACE2-Fc fusion (Mamedov et al., 2021c). Thus, production of recombinant proteins in a non-fused form has advantages over the same proteins with a fusion variant. It should be noted that the plant-produced S1 protein showed better stability compared with gRBD or dRBD (Mamedov et al., 2021a) and degraded by around 50% after 48 h and by 90% after 72 h. After two doses of plant-produced S1 protein, neutralizing titers of Omicron variant were reduced around eightfold compared with neutralizing titers of Wuhan; however, this reduction was not higher than fourfold for RBD or N+RBD counterparts (Mamedov et al., 2023). Considering that the expression level of the S1 protein is much higher than that of the RBD (Mamedov et al., 2021a) or N+RBD variants (Mamedov et al., 2021b), plant-produced S1 subunits of SARS-CoV-2 could be cost-effective and a safe protein-based vaccine candidate against COVID-19 variants.

## 5 Conclusion

Collectively, our results demonstrate that the plant-produced full-length S1 subunits could be potential promising vaccine candidates against COVID-19 variants. The amino acid sequence of the S1 protein used in this study corresponds to the 2020 Wuhan strain before the emergence of the SARS-CoV-2 variants including Delta and Omicron. Omicron is currently the dominant variant and is spreading worldwide. At this point, it is important to note that since the Omicron variant is characterized by significant changes in the spike protein and given the results observed in this study, the potential applicability of the study to the newest Omicron strain, EG.5, or informally “Eris,” could be considered. The observed lower reduction in antibody titers against Omicron, as compared with the Wuhan and Delta variants, suggests that targeting Omicron may indeed be a viable approach. It should be noted that while promising results have been observed in animal studies, further research and clinical trials are necessary to determine the safety, efficacy, and potential of any vaccine candidate, including those utilizing plant-based expression systems and targeting the S1 protein. Finally, the findings presented in this study are more important and relevant in the context of the current variants of SARS-CoV-2 and may provide potential directions for future research or therapeutic strategies.

## Data availability statement

The raw data supporting the conclusions of this article will be made available by the authors, without undue reservation.

## Ethics statement

The animal study was approved by Akdeniz University Experimental Animal Care Unit. The study was conducted in accordance with the local legislation and institutional requirements.

## Author contributions

TM: Conceptualization, Funding acquisition, Supervision, Writing – original draft, Writing – review & editing. DY: Investigation, Software, Writing – original draft. IG: Investigation, Writing – original draft. BG: Investigation, Writing – original draft. GM: Investigation, Writing – original draft. AO: Investigation, Writing – original draft. SP: Investigation, Writing – original draft. HY: Investigation, Writing – original draft, Data curation. BK: Writing – original draft, Investigation. MU: Investigation, Writing – original draft. GH: Writing – original draft, Writing – review & editing.
